# Infant body composition at 6 and 24 months: what are the driving factors?

**DOI:** 10.1038/s41430-023-01321-8

**Published:** 2023-08-10

**Authors:** Ina S. Santos, Caroline S. Costa, Andrew P. Hills, Shabina Ariff, V. Pujitha Wickramasinghe, Shane Norris, Alexia J. Murphy-Alford, Christine Slater, Nishani Lucas, Lukhanyo H. Nyati, Anura V. Kurpad, Kiran D. K. Ahuja, Rebecca Kuriyan, Multi-center Infant Body Composition Reference Study- MIBCRS, Ina S. Santos, Caroline S. Costa, Andrew P. Hills, Shabina Ariff, V. Pujitha Wickramasinghe, Shane Norris, Alexia J. Murphy-Alford, Christine Slater, Nishani Lucas, Anura V. Kurpad, Kiran D. K. Ahuja, Rebecca Kuriyan, Lukhanyo Nyati, Tanvir Ahmad, Jeffrey M. Beckett, Renata M. Bielemann, Nuala M. Byrne, Laila Charania, Michele P. Christian, Priscilla J. Divya, Anne Hanley, Manoja P. Herath, Leila C. Ismail, Sisitha Jayasinghe, Pulani Lanerolle, Cornelia Loechl, Najat Moktar, Upul Senerath, Sajid Soofi, Steven J. Street, Neiva C. J. Valle, Ayesha Yameen

**Affiliations:** 1 Federal University of Pelotas, Pelotas, Brazil; 2 University of Tasmania, Hobart, TAS Australia; 3 The Aga Khan University, Karachi, Pakistan; 4 University of Colombo, Colombo, Sri Lanka; 5 University of the Witwatersrand, Johannesburg, South Africa; 6 International Atomic Energy Agency, Vienna, Austria; 7 St John’s Research Institute, Bengaluru, India; 8 Isotope Application Division, Islamabad, Pakistan; 9 Institute of Nuclear Science and Technology (PINSTECH), Nilore, Pakistan; 10 University of Sharjah, Sharjah, United Arab Emirates

**Keywords:** Epidemiology, Risk factors

## Abstract

**Background/Objective:**

Available evidence on infant body composition is limited. This study aimed to investigate factors associated with body composition at 6 and 24 months.

**Subjects/Methods:**

Multicenter study with data from a 0 to 6-mo cohort (Australia, India and South Africa) and a 3 to 24-mo cohort (Brazil, Pakistan, South Africa, and Sri Lanka). For the 0–6-mo cohort, body composition was assessed by air-displacement plethysmography (ADP) and for the 3–24-month cohort by the deuterium dilution (DD) technique. Fat mass (FM), fat-free mass (FFM), FM index (FMI), and FFM index (FFMI) were calculated. Independent variables comprised the Gini index of the country, maternal and infant characteristics, and breastfeeding pattern at 3 months. For the 3–24-mo cohort, breastfeeding, and minimum dietary diversity (MDD) at 12 months were also included. Crude and adjusted analyses stratified by sex were conducted by multilevel modelling using mixed models.

**Results:**

At 6 months, every 1 kg increase in birth weight was associated with an increase of 0.716 kg in FFM and 0.582 kg/m^2^ in FFMI in girls, whereas in boys, the increase was of 0.277 kg in FFM. At 24 months, compared to those weaned before 12 months, girls still breastfed at 12 months presented a decrease of 0.225 kg in FM, 0.645 kg in FFM and 0.459 kg/m^2^ in FFMI, and in boys the decreases were of 0.467 kg in FM, 0.603 kg in FFM and 0.628 kg/m^2^ in FFMI.

**Conclusion:**

Birth weight and breastfeeding are independent predictors of body composition in early life, irrespective of sex.

## Introduction

Child malnutrition in all its forms, encompassing undernutrition and overweight, is a global public health problem with important consequences for survival, incidence of acute and chronic diseases, healthy development, and the economic productivity of individuals and societies [1]. The first 1000 days, from conception to a child’s second birthday, represent a critical period during which good nutrition and healthy growth have lasting benefits throughout life and are central for the concept of the developmental origins of chronic non-communicable diseases [2]. Gaining excess weight in early childhood potentially leads to increased adiposity and lifelong overweight and obesity and is associated with earlier onset of chronic diseases [3].

Growth assessment during early life has been mainly based on anthropometry. Nevertheless, infants with similar weight and length may vary substantially in the quality of growth and body composition [4]. Currently, the available data on body composition in early infancy is limited to single studies from a few countries. Thus, the objective of the present study was to investigate the factors associated with body composition at 6 and 24 months of age in a large cohort of children participating in a multicenter study.

## Methods

This was an observational, longitudinal, multinational study, that included data from two birth cohorts. The 0–6-mo cohort comprised infants from lower-middle (India), upper-middle (South Africa) and high-income (Australia) countries, and the 3–24-mo cohort enrolled children from lower-middle (Pakistan and Sri Lanka) and upper-middle (Brazil and South Africa) countries [5]. Participants were enrolled at birth at the Launceston General Hospital, in Launceston (Australia), at the five hospitals with maternity ward in Pelotas (Brazil), at St. John’s Medical College Hospital, Bangalore (India), at Aga Khan University Hospital in Karachi (Pakistan), at Chris Hani Baragwanath Academic Hospital in Johannesburg (South Africa), and at the University Unit of the De Soysa Hospital for Women, in Colombo (Sri Lanka). The eligibility criteria were a combination of those used in the World Health Organization (WHO) Multicentre Growth Reference Study (WHO-MGRS) and the INTERGROWTH-21st Project, aiming to select samples of newborns with no health, environmental or economic constraints on growth [6, 7]. Women admitted to hospitals were approached and screened by trained interviewers. Only mother-infant pairs that met all the eligibility criteria were invited to participate. The exclusion criteria followed at the study sites are depicted in Supplementary Table 1. More information on the study methodology can be found in another publication [8].

Sample size was calculated for the study sites to have a power of 90% to detect fat mass (FM) and fat-free mass (FFM) for boys and girls less than one standard deviation away from a reference study, that found mean FM 3.10 ± 0.5 kg and 3.05 ± 0.46 kg, and mean FFM 9.13 ± 1.06 kg and 8.99 ± 1.1 kg for boys and girls, respectively at 24 months of age [9].

For the 0–6-mo cohort, body composition was assessed by air-displacement plethysmography (ADP) (PEA POD, Software version 3.5.0, 201, COSMED, USA) at 6 months employing standard procedures [10]. Total body density was calculated as the ratio of weight (kg) and the measured body volume (L); proportions of FM and FFM were calculated using assumed densities (0.9007 and 1.063 kg/L for FM and FFM, respectively). For the 3–24-mo cohort, body composition was assessed using the deuterium dilution (DD) technique at 24 months of age. In this method, total body water (TBW) is measured from the dilution of a known quantity of deuterium oxide (D_2_O) in body water, which is evenly distributed throughout the body within a few hours and can be sampled in the form of saliva. D_2_O was administered and saliva samples were collected, stored, and analyzed according to standard procedures [11]. TBW was calculated using the weight of D_2_O consumed, the enrichment of the deuterium in the dose and the enrichment of deuterium in the saliva, with correction (4.1%) for non-aqueous exchange of deuterium [11]. FFM was estimated by dividing the estimated TBW by a sex and age-related constant for the hydration of FFM [12], and FM was calculated as the difference between body weight and FFM. For both cohorts, the parameters were expressed in kg (FM and FFM) and in kg/m^2^ (fat mass index—FMI, and fat-free mass index—FFMI).

Standardized protocols for anthropometry were developed based on the WHO-MGRS protocol [13] and used across all sites. For both cohorts, independent variables comprised the Gini index of the country, and maternal and infant characteristics. The Gini index is the most commonly used indicator of income distribution in a country [14], and measures the extent to which the distribution of income among individuals or households within an economy deviates from a perfectly equal distribution [15]. The index ranges between 0 and 100. A Gini index of 0 reflects perfect equality, where all income or wealth values are the same, while a Gini coefficient of 100 reflects maximal inequality among values.

Information on maternal age, years of schooling with approval, occupation (clerical support, service, or sales; housework; managerial, professional, or technical; skilled manual work; unskilled manual work; student; or other), marital status (married or cohabiting; and separated, divorced, or single), and mode of delivery (vaginal or cesarean section) were collected.

Variables from the infants included: sex (female, male), age (in months, calculated using date of birth and date of the last follow-up visit), gestational age in weeks, birth weight (BW), and birth length (for the 0–6-month cohort only), all obtained at the enrolment interview. Feeding practices included reported breastfeeding pattern at 3 months, collected at the 3-month follow-up. Breastfed infants that were not fed any other fluids, semi-solid or solid foods were classified as exclusively breastfed [16]. For the 3–24-mo cohort, continued breastfeeding and the minimum dietary diversity (MDD) were recorded at the 12-month follow-up, by means of 24 h dietary recall. MDD was considered present when the infant received foods from four or more food groups of the total seven groups (grains, roots and tubers; legumes and nuts; dairy products—milk, yogurt, cheese; flesh foods—meat, fish, poultry, and liver/organ meats; eggs; vitamin A rich fruits and vegetables; and other fruits and vegetables) [16].

### Statistical analyses

The sample characteristics at baseline were described for each country, according to the independent variables using means and proportions with 95% confidence interval (95% CI). Baseline characteristics were compared within and between countries. The follow-up rate at the end-line was calculated for each country, according to the independent variables. Due to the sample distribution and to avoid empty cells, maternal occupation was categorized as paid work and housework or student. Mean and 95% CI for each outcome were calculated jointly and for each country separately. Crude and adjusted beta coefficients (with 95% CI) for the outcomes were estimated using a mixed effects multilevel model, considering the natural clustering of data by country of the infant’s residence: the first level consisted of maternal and infant characteristics, and second level of the country. All variables were considered potential predictors of the outcomes and were entered and adjusted to each other in the adjusted model, attending the clustered nature of the data. As it has been recommended for the retention of confounding variables in a model [17], variables associated with the outcome with *p* ≤ 0.20 were retained in the multivariable model. Variables associated with the outcome at *p* > 0.20 were removed in backward fashion. All analyses were run separately for girls and boys with Stata 16.1® (StataCorp. 2019. Stata Statistical Software: Release 16. College Station, TX: StataCorp LLC). The statistical significance of differences between groups, and within categories of the same variable, was ascertained through the comparison of the range of values of the 95% CI of estimates. Statistical significance level was two-tailed and set at *p* < 0.05.

## Results

A total of 468 newborns were included in the 0–6-mo cohort (Australia = 133, India = 102, and South Africa = 233), 39.7% of whom were followed-up at 6 months (Australia = 85, India = 96, and South Africa = 50) (at 6 months, the majority of infants from South Africa were over 8 kg, exceeding the capacity of the equipment). For the 3–24-mo cohort, 925 newborns were included (Brazil = 293, Pakistan = 143, South Africa = 398, and Sri Lanka = 91), of whom 490 (53%) were assessed at the 24-month follow-up (Brazil = 211, Pakistan = 113, South Africa = 141, and Sri Lanka = 25). Supplementary Tables 2 and 3 presents, respectively, the description of the 0–6-mo and 3–24-mo cohort at the inception and at endline. In both cohorts, there was no difference in maternal and infant characteristics at birth between infants who were followed-up at endline and those who were not.

Taken together, the boys had higher FFM than girls at 6 (5.55 kg versus 5.09 kg) (Table 1) and 24 months (9.41 kg versus 9.03 kg) (Table 2). All other indices, including FFMI, were similar between boys and girls at both ages.

**Table 1:** Means with 95% CIs for anthropometric and body composition indices at 6 months of age, stratified by sex, among infants from the 0 to 6-mo cohort.

Outcomes	Australia	India	South Africa	All countries
*Girls*	*n* = 45	*n* = 48	*n* = 31	*n* = 124
Weight, kg	7.11 (6.93; 7.30)	6.78 (6.56; 7.00)	7.21 (6.97; 7.44)	7.01 (6.89; 7.13)
Length, cm	64.55 (63.95; 65.15)	65.20 (64.56; 65.84)	64.08 (63.17; 64.99)	64.68 (64.28; 65.08)
FM, kg	1.89 (1.78; 2.00)	1.87 (1.72; 2.02)	2.02 (1.84; 2.20)	1.92 (1.84; 2.00)
FFM, kg	5.22 (5.07; 5.37)	4.91 (4.79; 5.04)	5.17 (5.04; 5.31)	5.09 (5.01; 5.17)
FMI, kg/m^2^	4.55 (4.28; 4.81)	4.38 (4.07; 4.70)	4.99 (4.48; 5.50)	4.59 (4.39; 4.79)
FFMI, kg/m^2^	12.51 (12.27; 12.76)	11.55 (11.33; 11.77)	12.65 (12.20; 13.10)	12.17 (11.99; 12.36)

*Boys*	*n* = 40	*n* = 48	*n* = 19	*n* = 107
Weight, kg	7.43 (7.21; 7.65)	7.52 (7.30; 7.74)	7.36 (7.00; 7.73)	7.46 (7.32; 7.60)
Length, cm	66.33 (65.65; 67.01)	66.87 (66.30; 67.45)	66.44 (65.15; 67.74)	66.59 (66.18; 67.01)
FM, kg	1.79 (1.67; 1.91)	2.01 (1.85; 2.17)	1.91 (1.68; 2.14)	1.91 (1.82; 2.00)
FFM, kg	5.65 (5.49; 5.80)	5.51 (5.38; 5.64)	5.45 (5.26; 5.65)	5.55 (5.46; 5.64)
FMI, kg/m^2^	4.06 (3.79; 4.34)	4.48 (4.15; 4.82)	4.40 (3.81; 4.98)	4.31 (4.11; 4.52)
FFMI, kg/m^2^	12.83 (12.57; 13.08)	12.32 (12.06; 12.59)	12.42 (11.90; 12.94)	12.53 (12.35; 12.71)

**Table 2:** Means with 95% CIs for anthropometric and body composition indices at 24 months of age, stratified by sex, among infants from the 3 to 24-month cohort.

Outcomes	Brazil	Pakistan	South Africa	Sri Lanka	All countries
*Girls*	*n* = 106	*n* = 58	*n* = 70	*n* = 12	*n* = 246
Weight, kg	12.12 (11.83; 12.40)	11.18 (10.82; 11.53)	11.25 (10.89; 11.61)	11.03 (10.41; 11.64)	11.60 (11.41; 11.78)
Length, cm	86.86 (86.19; 87.53)	85.75 (84.90; 86.59)	83.61 (82.80; 84.38)	85.29 (83.75; 86.83)	85.60 (85.15; 86.04)
FM, kg	2.67 (2.54; 2.80)	1.88 (1.72; 2.04)	3.16 (2.96; 3.36)	1.73 (1.37; 2.10)	2.58 (2.47; 2.69)
FFM, kg	9.45 (9.22; 9.68)	9.33 (9.03; 9.63)	11.57 (11.33; 11.82)	12.76 (12.31; 13.20)	9.03 (8.87; 9.19)
FMI, kg/m^2^	3.53 (3.37; 3.69)	2.56 (2.35; 2.76)	4.50 (4.24; 4.76)	2.36 (1.89; 2.83)	3.52 (3.37; 3.67)
FFMI, kg/m^2^	12.49 (12.29; 12.69)	12.68 (12.36; 13.00)	11.57 (11.33; 11.82)	12.76 (12.31; 13.20)	12.29 (12.14; 12.43)

*Boys*	*n* = 105	*n* = 55	*n* = 71	*n* = 13	*n* = 244
Weight, kg	12.76 (12.45; 13.07)	11.21 (10.81; 11.60)	11.82 (11.47; 12.18)	11.02 (10.39; 11.65)	12.04 (11.84; 12.25)
Length, cm	88.40 (87.78; 89.02)	85.11 (84.19; 86.03)	84.74 (83.82; 85.65)	85.62 (83.60; 87.64)	86.44 (85.96; 86.93)
FM, kg	2.60 (2.43; 2.77)	2.42 (2.20; 2.65)	3.07 (2.87; 3.27)	1.57 (1.14; 2.00)	2.64 (2.52; 2.76)
FFM, kg	10.19 (9.97; 10.40)	8.79 (8.49; 9.08)	8.73 (8.46; 8.99)	9.47 (8.66; 10.28)	9.41 (9.24; 9.57)
FMI, kg/m^2^	3.31 (3.11; 3.51)	3.33 (3.04; 3.61)	4.26 (4.00; 4.52)	2.15 (1.55; 2.75)	3.53 (3.38; 3.68)
FFMI, kg/m^2^	13.02 (12.81; 13.22)	12.10 (11.82; 12.38)	12.14 (11.86; 12.42)	12.90 (11.94; 13.87)	12.55 (12.40; 12.70)

Crude and adjusted beta coefficients for FM, FFM, FMI, and FFMI among girls and boys at 6 months according to the independent variables are shown in Table 3. The crude and adjusted beta coefficients at 24 months, for girls and boys, are shown in Table 4. The Tables 3 and 4 present exclusively the variables that were statistically associated with at least one of the body composition parameters in the adjusted analyses.

**Table 3:** Crude and adjusted beta coefficients for body composition indices, according to independent variables, among infants from the 0 to 6-month cohort.

	FM			FFM			FMI			FFMI		
	β crude (95% CI)	β adjusted (95% CI)	*P*	β crude (95% CI)	β adjusted (95% CI)	*P*	β crude (95% CI)	β adjusted (95% CI)	*P*	β crude (95% CI)	β adjusted (95% CI)	*P*
*GIRLS*												
*Maternal characteristics*												
Marital status												
Married or cohabiting	Ref	Ref	–	Ref	Ref	0.035	Ref	Ref	–	Ref	Ref	–
Separated, divorced, or single	0.092 (−0.095; 0.280)	–		0.007 (−0.261; 0.274)	−0.420 (−0.812; −0.028)		0.401 (−0.053; 0.855)	–		0.176 (−0.478; 0.830)	–	
Delivery mode						–						
Vaginal	Ref	Ref	–	Ref	Ref	0.029	Ref	Ref	–	Ref	Ref	–
Cesarean section	0.029 (−0.173; 0.231)	–		0.094 (−0.110; 0.297)	0.069 (0.007; 0.131)		−0.010 (−0.503; 0.482)	–		0.123 (−0.296; 0.541)	–	
*Infant characteristics at birth*												
Birth weight, kg	0.053 (−0.145; 0.251)	–	–	0.624 (0.450; 0.797)	0.716 (0.517; 0.914)	<0.001	−0.072 (−0.546; 0.402)	–	–	0.547 (0.058; 1.035)	0.582 (0.143; 1.020)	0.009
*Feeding practices*												
Exclusive breastfeeding at 3 mo												
No	Ref	Ref	–	Ref	Ref	0.062	Ref	Ref	0.051	Ref	Ref	<0.001
Yes	−0.105 (−0.277; 0.067)	–		−0.157(−0.344;0.030)	−0.194(−0.397;0.009)		−0.415(−0.830;0.001)	−0.415(−0.830;0.001)		−0.632 (−1.020; −0.245)	−0.700 (−1.076; −0.325)	
*BOYS*												
*Maternal characteristics*												
Age, years	−0.016 (−0.033;0.001)	−0.016 (−0.033;0.001)	0.070	−0.022 (−0.039;-0.005)	−0.028 (−0.045;−0.011)	0.001	−0.025 (−0.064;0.013)	–	–	−0.027 (−0.061;0.008)	−0.045 (−0.078;−0.012)	0.008
Schooling, y	−0.026 (−0.062;0.010)	–	–	0.022 (−0.012;0.055)	0.049 (0.012;0.086)	0.009	−0.068 (−0.146;0.011)	−0.068 (−0.146;0.011)	0.092	0.008 (−0.064;0.080)	–	–
Delivery mode												
Vaginal	Ref	Ref	–	Ref	Ref	–	Ref	Ref	–	Ref	Ref	0.001
Caesarean section	−0.024 (−0.237;0.190)	–		0.165 (−0.037;0.367)	–		0.017 (−0.461;0.495)	–		0.620 (0.225;1.015)	0.224 (0.098;0.351)	
*Infant characteristics at birth*												
Birth weight, kg	0.158 (−0.095;0.411)	–	–	0.306 (0.102;0.511)	0.277(0.067;0.487)	0.010	0.227 (−0.325;0.779)	–		0.617 (0.197;1.037)	–	
*Feeding practices*												
Exclusive breastfeeding at 3 mo												
No	Ref	Ref	–	Ref	Ref	0.042	Ref	Ref	–	Ref	Ref	0.002
Yes	0.024 (−0.199;0.246)	–		−0.186 (−0.407;0.035)	−0.202 (−0.396;-0.007)		−0.004 (−0.493;0.486)	–		−0.584 (−1.036;−0.132)	−0.712 (−1.165;−0.258)

**Table 4:** Crude and adjusted beta coefficients for body composition indices at 24 months of age among children from the 3 to 24-month cohort.

	FM			FFM			FMI			FFMI		
	β crude (95%CI)	β adjusted (95%CI)	*p*	β crude (95%CI)	β adjusted (95%CI)	*p*	β crude (95%CI)	β adjusted (95%CI)	*p*	β crude (95%CI)	β adjusted (95%CI)	*p*
*GIRLS*												
Contextual variable												
Gini index	0.042 (0.034;0.049)	0.042 (0.034;0.050)	<0.001	−0.032 (−0.065;0.002)	−0.014 (−0.030;0.002)	0.079	0.063 (0.045;0.081)	0.063 (0.053;0.074)	<0.001	−0.032 (−0.054;−0.010)	−0.025 (−0.040;−0.009)	0.002
*Maternal characteristics*												
Marital status									–			
Married or cohabiting	Ref	Ref	–	Ref	Ref	<0.001	Ref	Ref		Ref	Ref	<0.001
Separated, divorced, or single	0.393 (−0.016;0.802)	–		−1.246 (−1.561;−0.932)	−1.174 (−1.592;−0.757)		0.467 (−0.065;1.000)	–		−1.053 (−1.352;−0.753)	−0.789 (−1.207;−0.372)	
*Child characteristics at birth*												
Birth weight, kg	0.318 (0.100;0.535)	0.312 (0.081;0.544)	0.008	0.739 (0.408;1.070)	0.834 (0.510;1.159)	<0.001	0.278 (−0.002;0.558)	–	–	0.483 (0.153;0.813)	0.518 (0.182;0.854)	0.003
*Feeding practices*												
Continued breastfeeding at 12 mo												
No	Ref	Ref	0.044	Ref	Ref	<0.001	Ref	Ref	–	Ref	Ref	0.005
Yes	−0.127 (−0.354;0.099)	−0.225 (−0.444;−0.006)		−0.554 (−0.894;−0.215)	−0.645 (−0.959;−0.331)		−0.079 (−0.368;0.210)			−0.454 (−0.792;−0.115)	−0.459 (−0.780;−0.137)	
Minimum dietary diversity at 12 mo												
No	Ref	Ref	0.005	Ref	Ref	0.073	Ref	Ref	0.031	Ref	Ref	–
Yes	−0.256 (−0.475;−0.036)	−0.300 (−0.508;−0.092)		−0.258 (−0.599;0.084)	−0.281 (−0.589;0.027)		−0.243 (−0.523;0.037)	−0.294 (−0.561;−0.028)		0.010 (−0.331;0.351)	–	
*BOYS*												
*Maternal characteristics*												
Occupation												
Paid work	Ref	Ref	–	Ref	Ref	0.045	Ref	Ref	0.001	Ref	Ref	0.185
Housework/Student	−0.108 (−0.389;0.173)	–		−0.577 (−0.937;−0.217)	−0.363 (−0.719;−0.008)		−0.028 (−0.375;0.320)	0.741 (0.314;1.167)		−0.358 (−0.716;−0.001)	−0.250 (−0.619;−0.119)	
*Child characteristics at birth*												
Gestational age, wks	0.034 (−0.063;0.131)	0.069 (−0.035;0.174)	0.194	−0.075 (−0.202;0.053)	−0.139 (−0.273;−0.005)	0.042	0.046 (−0.075;0.167)	0.189 (0.026;0.353)	0.023	−0.106 (−0.234;0.023)	−0.232 (−0.394;−0.070)	0.005
Birth weight, kg	0.246 (−0.018;0.511)	0.191 (−0.075;0.456)	0.159	1.010 (0.685;1.335)	1.061 (0.720;1.402)	<0.001	0.138 (−0.192;0.468)	–	–	0.690 (0.352;1.027)	0.972 (0.576;1.368)	<0.001
*Feeding practices*												
Continued breastfeeding at 12 mo												
No	Ref	Ref	0.001	Ref	Ref	0.001	Ref	Ref	0.063	Ref	Ref	0.002
Yes	−0.378 (−0.638;−0.117)	−0.467 (−0.732;−0.203)		−0.563 (−0.927;−0.198)	−0.603 (−0.947;−0.259)		−0.408 (−0.736;−0.080)	−0.373 (−0.765;0.020)		−0.537 (−0.899;−0.175)	−0.628 (−1.030;−0.227)	
Minimum dietary diversity at 12 mo												
No	Ref	Ref	–	Ref	Ref	0.008	Ref	Ref	–	Ref	Ref	0.009
Yes	0.086 (−0.196;0.368)	–		0.306 (−0.087;0.700)	0.494 (0.129;0.860)		0.079 (−0.274;0.432)	–		0.266 (−0.123;0.655)	0.543 (0.133;0.953)	

### Body composition at 6 months

In adjusted analyses, maternal age and schooling were related to body composition only among boys, whereas maternal marital status was associated only among girls. A 1-year increase in maternal age was associated with a decrease of 0.028 kg in FFM and 0.045 kg/m^2^ in FMI, whereas a 1-year increase in maternal schooling increased 0.049 kg in FFM. FFM of girls born to separated/divorced/single mothers was on average 0.420 kg lower than in their counterparts. FFM was on average 0.069 kg higher in girls born by caesarean section than in those born via vaginal. In boys, caesarean section was related to an increase of 0. 224 kg/m^2^ in FFMI. Maternal work, the Gini index of the country and gestational age were not associated with any of the outcomes at 6 months in both sexes.

BW was associated with FFM in boys and girls and FFMI in girls. In girls, a 1 kg increase in BW was associated with an increase of 0.716 kg in FFM and 0.582 kg/m^2^ in FFMI. In boys, every kg in BW was associated with an increase of 0.277 kg in FFM. In comparison to their counterparts, exclusively breastfed girls at 3 months presented a reduction of 0.700 kg/m^2^ in FFMI at 6 months; in boys, the reduction was of 0.202 kg in FFM and 0.712 kg/m^2^ in FFMI.

### Body composition at 24 months

The body composition of girls, but not boys at 24 months was related to the Gini index of the country and to maternal marital status. The increase in the Gini index was associated with an increase of 0.042 kg in FM, 0.063 kg/m^2^ in FMI and a decrease of 0.025 kg/m^2^ in FFMI. Girls born to separated, divorced, or single mother had a decrease of 1.174 kg in FFM and 0.789 kg/m^2^ in FFMI in comparison to those from married or cohabiting mothers. Maternal work was associated with body composition at 24 months only among boys. FFM and FMI of boys from housework/student mothers were on average 0.363 kg and 0.741 kg/m^2^ lower than in those from mothers in paid work. Maternal age, schooling, type of delivery, and exclusive breastfeeding at 3 months were not related to any of the outcomes at 24 months in both sexes.

Gestational age was associated with body composition at 24 months only in boys. Every 1-week increase in gestational age was associated with an increase of 0.189 kg/m^2^ in FMI and a decrease of 0.139 kg in FFM and 0.232 kg/m^2^ in FFMI. In girls and boys, FFM and FFMI increased with the increase in BW. In girls, every increase of 1 kg in BW was associated with an increase of 0.834 kg in FFM and 0.518 kg/m^2^ in FFMI. In boys the increase was of 1.061 kg in FFM and 0.972 kg/m^2^ in FFMI. In girls, BW was also related to FM with every increase of 1 kg in BW being associated with an increase of 0.312 kg in FM.

Continued breastfeeding at 12 months reduced FM, FFM and FFMI in boys and girls. Compared to those weaned before 12 months, girls still breastfed at 12 months presented a decrease of 0.225 kg in FM, 0.645 kg in FFM and 0.459 kg/m^2^ in FFMI. In boys still breastfed at 12 months the decreases were of 0.467 kg in FM, 0.603 kg in FFM and 0.628 kg/m^2^ in FFMI. Girls with MDD presented 0.300 kg less in FM than those without MDD at 12 months. Among boys with MDD there was an increase of 0.494 kg in FFM and 0.543 kg/m^2^ in FFMI, in comparison to those without MDD at 12 months.

## Discussion

To our knowledge this is the first multinational study that accurately measured body composition in many countries, including low- and middle-income countries (LMICs). This study showed that in this specific population of “better-off” participants, variance in body composition at 6 months, mainly FFM, is partly explained by BW, clearly indicating the importance of the pregnancy period and fetal growth. At 24 months, the association of BW with FFM still persists, despite the greater environmental influence represented by continued breastfeeding and diversity of diet.

BW was the most consistent positive predictor of FFM at 6 and 24 months irrespective of sex. Our findings are in agreement with several studies that used a variety of measurement techniques to explore the relationship between BW and FFM [18, 19]. The direct association between BW and FFM is present across populations and is apparent across the lifespan; whereas the evidence is less consistent for body fat, with studies finding negative, positive or non-significant associations [18], even when the presence of intra-uterine growth restriction is considered [20, 21]. In our study, BW in girls was also related to higher FM at 24 months. In a previous study, sex difference in the association of BW with FM was observed only in girls at 9 years of age [22].

We found a decrease in FMI at 6 months in boys and girls who received exclusive breastfeeding at 3 months (a decrease in FFM was also present in boys), whereas Butte et al. [23] reported lower adiposity in the first 3–6 months of life in exclusively formula-fed, as compared to exclusively breastfed infants. A meta-analysis to explore the association between breastfeeding and childhood obesity found that the risk of obesity was lower in breastfed children by 22% compared with those who were never breastfed [24].

Continued breastfeeding at 12 months was associated with lower body composition indices at 24 months in both boys and girls. A systematic review summarizing the postnatal biological determinants of early weight gain related to infant feeding reported that breastfed infants exhibit different body composition trajectories than formula-fed infants during the first 6 months [25]. Formula-fed babies have higher FFM throughout the first year of life than breastfed babies but changes in FM over this period are more complex, with formula-fed babies having lower FM than their breastfed counterparts at 3–4 and 6 months [26]. Conversely, at 12 months, FM was higher in formula-fed infants than in breastfed infants [27].

Complementary foods are necessary to meet nutritional needs of the infant after 6 months of age along with breastfeeding. In our study, at 24 months MDD measured at 12 months was positively related to FFM and FFMI only in boys (in girls, MDD was negatively associated with FM). A cross-sectional study with 6–8 month old infants in Senegal found that MDD was significantly associated with high body fat and percent body fat but not with variation of FFM or FFMI [28].

At 6 months, caesarean section was positively associated with FFM in girls and FFMI in boys. Nonetheless, these results should be considered with caution, because most of the deliveries by caesarean section were from Brazil, a country where this mode of delivery is strongly associated with the wealthiest family conditions [29].

Due to the eligibility criteria, all infants in our study were at term (37–41 weeks of gestational age). Despite this, we found a negative relationship of gestational age with FFM and a positive association with FMI in boys at 24 months of age.

Among girls, the Gini index of the country was positively related to FM and FMI and negatively related to FFMI at 6 months. In high-income countries, several studies have reported associations between disadvantaged socioeconomic position and higher FM among girls, compared to boys [30]. There were no papers from low-income countries, limiting the ability to explore socioeconomic position and body composition associations in countries at an earlier stage of the nutrition transition [30]. There is evidence, however, that the burden of infant malnutrition is greatest in LMICs [31] and that its determinants comprise maternal nutrition, postnatal diet, disease, and nurturing care, which are, in turn, affected by distal socioeconomic (including maternal education, paternal education, household assets, and early marriage) and political factors [32, 33]. In addition to driving within-country inequalities, socioeconomic factors also account for a large proportion of between-country variability in undernutrition [34]. Nonetheless nutritional interventions with potential to decrease stunting, based on evidence from outside of slum contexts, fail to preventing stunting in children living in urban slums in LMICs [35]. At least three reasons may be involved in the failure of nutritional interventions in LMIC settings. First, stunting as well as wasting may already be present at birth (with the incidence of both conditions peaking in the first 6 months of life) [34], and the development is characterized by a succession of sensitive periods (critical windows), when phenotype is particularly responsive to nutritional influences [36]. Many critical windows close early during development, reducing the sensitivity of specific traits to environmental influences. For instance, from late infancy linear growth becomes less sensitive to nutritional intake, hence the environmental contribution to short adult stature is primarily attributable to early stunting [36]. Second, deficiencies in implementation when delivered under real conditions reduce the effectiveness of interventions that have proven to be effective when implemented under ideal conditions [37]. And third, there is evidence that stunting, as defined by the World Health Organization standards [38], is not synonym for chronic malnutrition [39]. Although better nutrition leads to less stunting, Scheffler et al., in a study with 1716 Indonesian children, aged 6.0–13.2 years, showed that stunted children were not uniformly characterized by depleted fat stores; fat stores of less stunted children were not less depleted; better parental education did not minimize the risk of child undernutrition; and stunted children did not exhibit visible clinical signs of protein-energy malnutrition [39].

In addition, a study aiming to investigate the association of body weight and weight variability among populations from different geographic, historic, and socioeconomic background found that prosperous conditions lead to growth improvements in height and weight at a large scale (wealthy countries have tall and heavy children), whereas at small scale, the discrepancy between the within-population variation in height and weight strongly suggests that height gains and weight gains are subject to different regulations [40]. Although wasting and stunting share common determinants in utero and in infancy, there is evidence that wasting increases the risk of subsequent stunting, suggesting that the body responds to weight faltering by slowing linear growth [41]. Wasted children have a high risk of dying, which can often be rapidly reduced by nutritional therapy [42], whereas growth faltering in stunted children (due to poor growth in height over long periods, including fetal development) is not amenable to rapid nutritional correction and requires prevention rather than treatment [43].

The FFM of girls born to separated/divorced/single mothers in the present study were lower than in those whose mother lived with a partner. This finding suggests social and economic impact on infant growth. Chronic and acute undernutrition are primarily associated with inequalities in financial and material resources between households and nations [44], and the absence of one parent in a family reduces time resource available for monitoring and supervision of children (combining work and parenting not only affects the time a single mother has for childcare, but it also introduces stress that negatively affects her psychological well-being and parenting effectiveness) [45].

Although older mothers tend to be heavier and their newborns tend to be larger [46], we found no association between maternal age and FM or FMI in boys and girls from both cohorts. In fact, there was a negative association between maternal age, FFM and FFMI at the 0–6-month cohort among boys only, thus indicating that the older the mother the lower the amount of FFM and the lower the FFMI. A previous study found that preventable gestational factors (like greater gestational weight gain, gestational arterial hypertension, and gestational diabetes) were more influential on the greater amount of neonatal FM, whereas demographic characteristics (maternal age, gestational age, and newborn’s gender) contributed to the lower FFM in newborns [47].

### Strengths and limitations

The strengths of this study include the recruitment of newborns with no health, environmental or economic constraints on growth, using the same entry criteria, equipment, data collection methods, and standardization procedures across countries. The participation of six countries from four distinct continents increases the external validity of our findings. Moreover, body composition was measured longitudinally, using accurate methods, in a relatively large cohort where most infants were exclusively breastfed for at least 3 months. On the other hand, as heavy-smoking mothers were not eligible to the study, the potential association between intra-uterine exposure to tobacco and body composition could not be explored. In the same way, maternal pre-pregnancy body mass index and gestational weight gain were not collected and could not be investigated. Also, information on other dietary data and illness of the child were not available.

## Conclusions

The main results of this study were the consistent association of BW and infant feeding with body composition for both sexes. Higher BW was associated with higher FFM at 6 and 24 months and continued breastfeeding at 12 months was associated with lower FM, FFM and FFMI at 24 months. MDD at 12 months was associated with lower FM in girls, and higher FFM and FFMI in boys. Our findings support continued breastfeeding and MDD at 12 months as strategies to promote healthy body composition in early life.

## Supplementary information

Supplementary material

## Data Availability

Data described in the paper, code book, and analytic code will be made publicly and freely available without restriction at https://witscloud-my.sharepoint.com/personal/00000085_wits_ac_za/_layouts/15/onedrive.aspx?id=%2Fpersonal%2F00000085%5Fwits%5Fac%5Fza%2FDocuments%2FIAEA%20MIBCRS%20Data&ga=1.
