# Correction: Infant body composition at 6 and 24 months: what are the driving factors?

**DOI:** 10.1038/s41430-023-01346-z

**Published:** 2023-12-01

**Authors:** Ina S. Santos, Caroline S. Costa, Andrew P. Hills, Shabina Ariff, V. Pujitha Wickramasinghe, Shane Norris, Alexia J. Murphy-Alford, Christine Slater, Nishani Lucas, Lukhanyo H. Nyati, Anura V. Kurpad, Kiran D. K. Ahuja, Rebecca Kuriyan, Multi-center Infant Body Composition Reference Study- MIBCRS, Ina S. Santos, Caroline S. Costa, Andrew P. Hills, Shabina Ariff, V. Pujitha Wickramasinghe, Shane Norris, Alexia J. Murphy-Alford, Christine Slater, Nishani Lucas, Anura V. Kurpad, Kiran D. K. Ahuja, Rebecca Kuriyan, Lukhanyo Nyati, Tanvir Ahmad, Jeffrey M. Beckett, Renata M. Bielemann, Nuala M. Byrne, Laila Charania, Michele P. Christian, Priscilla J. Divya, Anne Hanley, Manoja P. Herath, Leila C. Ismail, Sisitha Jayasinghe, Pulani Lanerolle, Cornelia Loechl, Najat Moktar, Upul Senerath, Sajid Soofi, Steven J. Street, Neiva C. J. Valle, Ayesha Yameen

**Affiliations:** 1 Federal University of Pelotas, Pelotas, Brazil; 2 University of Tasmania, Hobart, TAS Australia; 3 The Aga Khan University, Karachi, Pakistan; 4 University of Colombo, Colombo, Sri Lanka; 5 University of the Witwatersrand, Johannesburg, South Africa; 6 International Atomic Energy Agency, Vienna, Austria; 7 St John’s Research Institute, Bengaluru, India; 8 Isotope Application Division, Islamabad, Pakistan; 9 Institute of Nuclear Science and Technology (PINSTECH), Nilore, Pakistan; 10 University of Sharjah, Sharjah, United Arab Emirates

**Keywords:** Epidemiology, Risk factors

Correction to: *European Journal of Clinical Nutrition* 10.1038/s41430-023-01321-8, published online 10 August 2023

Two of the authors in the MIBCRS consortium list were assigned incorrect affiliations. The affiliation for Manoja P Herath and Steven Street has been corrected to ‘University of Tasmania, Hobart, TAS, Australia’. The original article has been corrected.

