# Supplementary material for: Infant body composition at 6 and 24 months: what are the driving factors?
Source: Eur J Clin Nutr. 2023 Aug 10;78(11):928–35. doi: 10.1038/s41430-023-01321-8 (PMC11537963; doi:10.1038/s41430-023-01321-8)
Supplement: Supplementary file 1 — Supplementary material [file 41430_2023_1321_MOESM1_ESM.docx]

**INFANT BODY COMPOSITION AT 6 AND 24 MONTHS: WHAT ARE THE DRIVING FACTORS?**

**SUPLEMMENTARY MATERIAL**

Supplementary Table 1. Exclusion criteria for the Multi-Centre Body Composition Study.

Supplementary Table 2. Sample distribution at birth and at the 6-month follow-up (with 95% confidence interval), according to maternal and infant characteristics, among infants from the 0-6-month cohort.

Supplementary Table 3. Sample distribution at birth and at the 24-month follow-up (with 95% confidence interval), according to maternal and child characteristics, among infants from the 3-24-month cohort.

| **Supplementary Table 1.** Exclusion criteria for the Multi-Centre Body Composition Study. | |
| --- | --- |
| **Exclusion criteria** | **Description** |
| Family income | Low family monthly income |
| Living area | Mother living outside the study area of each site |
| Feeding intentions and exclusive breastfeeding | - Mother is not willing to try exclusive breastfeeding for the first 6 months |
|  | - Mother is not willing to breastfeed for at least 12 months |
|  | - Mother will not breastfeed |
| Gestational age* | Unknown gestational age; infants born before 37^+0^ or after 41^+6^ weeks |
| Maternal age | < 18 years old |
| Maternal smoking | “Heavy” smoker (four days a week or more during pregnancy and intention to smoke four days a week or more after childbirth) |
| Newborn morbidity | Newborn admitted to intensive care or any special care unit or presence of morphological abnormality |
| Pregnancy | Multiple pregnancy |

*^*^ Gestational age based in the best dating information available. First preference was crown-rump length (CRL) measurement taken before 14 weeks; second preference was head circumference measurement taken before 24 weeks of pregnancy; and third preference was the first day of the mother last menstrual period (LMP).*

| **Supplementary Table 2. Sample distribution at birth and at the 6-month follow-up (with 95% confidence interval), according to maternal and infant characteristics, among infants from the 0-6-month cohort.** | | | | | | | |
| --- | --- | --- | --- | --- | --- | --- | --- |
|  | **At birth** | | |  | **At 6-mo follow-up** | | |
|  | **Australia**  **(*n* = 133)** | **India**  **(*n* = 102)** | **South Africa**  **(*n* = 233)** |  | **Australia**  **(*n* = 85)** | **India**  **(*n* = 96)** | **South Africa**  **(*n* = 50)** |
| ***Maternal characteristics*** |  |  |  |  |  |  |  |
| Age in y, mean | 30.5  (29.7; 31.4) | 26.4  (25.4; 27.4) | 26.2  (25.0; 27.3) |  | 30.7  (29.7; 31.7) | 28.6  (27.7; 29.4) | 26.5  (25.7; 27.4) |
| Schooling in y, mean | 14.9  (14.5; 15.3) | 13.3  (12.8; 13.8) | 12.0  (11.9; 12.1) |  | 14.9  (14.3; 15.6) | 14.3  (13.8; 14.9) | 11.9  (11.8; 12.1) |
| Occupation, (%) |  |  |  |  |  |  |  |
| Paid work | 87.2 (80.3; 91.9) | 15.0  (9.2; 23.5) | 74.1  (63.6; 82.4) |  | 85.9  (76.6; 91.9) | 16.0  (9.8; 24.9) | 72.4  (52.8; 86.0) |
| Housework/ Student | 12.8 (8.1; 19.7) | 85.0  (76.5; 90.8) | 25.9  (17.6; 36.4) |  | 14.1  (8.1; 23.4) | 84.0  (75.1; 0.2) | 27.6  (14.0; 47.2) |
| Marital status, (%) |  |  |  |  |  |  |  |
| Married or cohabiting | 94.7  (89.3; 97.5) | 100  (100; 100) | 22.4  (15.7; 31.0) |  | 92.9  (84.8; 96.8) | 100  (100; 100) | 22.2  (9.9; 42.5) |
| Delivery mode, (%) | |  |  |  |  |  |  |
| Vaginal | 70.7  (62.3; 77.8) | 71.0  (61.3; 79.1) | 100  (100; 100) |  | 71.4  (60.7; 80.2) | 72.9  (62.4; 81.4) | 100  (100; 100) |
| ***Infant characteristics*** |  |  |  |  |  |  |  |
| Sex, (%) |  |  |  |  |  |  |  |
| Female | 50.4  (41.9; 58.9) | 48.0  (38.4; 57.8) | 51.9  (45.5; 58.3) |  | 52.4  (41.6; 62.9) | 52.9  (42.2; 63.4) | 66.7  (46.4; 82.2) |
| Male | 49.6  (41.1; 58.1) | 52.0  (42.2; 61.6) | 48.1  (41.7; 54.5) |  | 47.6  (37.1; 58.4) | 47.1  (36.6; 57.8) | 33.3  (17.8; 53.6) |
| Gestational age in wk, mean | 39.6  (39.4; 39.8) | 38.9  (38.7; 39.0) | 39.0  (38.8; 39.3) |  | 39.8  (39.6; 40.1) | 38.9  (38.7; 39.1) | 39.3  (38.8; 39.7) |
| Birthweight in kg, mean | 3,337  (3,264; 3,410) | 2,801  (2,741; 2,862) | 3,052  (3,002; 3,103) |  | 3,324  (3,238; 3,409) | 2,794  (2,731; 2,856) | 2,965  (2,874-3,056) |
| Length, cm | 49.9  (49.6; 50.2) | 48.4  (48.0; 48.7) | 48.1  (47.9; 48.3) |  | 49.7  (49.3; 50.1) | 48.4  (48.1; 48.7) | 47.8  (47.3; 48.3) |
| ***Feeding practices*** |  |  |  |  |  |  |  |
| Exclusive breastfeeding at 3 mo, (%) | - | - | - |  | 70.2  (59.5; 79.1) | 94.1  (86.5-; 97.6) | 18.5  (7.6; 38.6) |

| **Supplementary Table 3. Sample distribution at birth and at the 24-month follow-up (with 95% confidence interval), according to maternal and child characteristics, among infants from the 3-24-month cohort.** | | | | | | | | | |
| --- | --- | --- | --- | --- | --- | --- | --- | --- | --- |
|  | **At birth** | | | |  | **At 24-month follow-up** | | | |
|  | **Brazil**  **(*n* = 293)** | **Pakistan**  **(*n* = 143)** | **South Africa**  **(*n* = 398)** | **Sri Lanka**  **(*n* = 91)** |  | **Brazil**  **(*n* = 211)** | **Pakistan**  **(*n* = 113)** | **South Africa**  **(*n* = 141)** | **Sri Lanka**  **(*n* = 25)** |
| ***Maternal characteristics*** | |  |  |  |  |  |  |  |  |
| **Age in years, mean** | 30.1  (29.5; 30.8) | 28.4  (27.6; 29.1) | 26.4  (25.9; 27.0) | 29.9  (28.7; 31.2) |  | 30.5  (29.7; 31.2) | 28.6  (27.7; 29.4) | 26.5  (25.7-27.4) | 32.8  (30.3; 35.3) |
| **Schooling in years, mean** | 14.1  (13.6; 14.5) | 14.2  (13.7; 14.6) | 12.0  (11.9; 12.1) | 11.6  (11.2; 12.0) |  | 14.0  (13.6; 14.5) | 14.3  (13.8; 14.9) | 11.9  (11.8; 12.1) | 11.5  (10.8; 12.2) |
| **Occupation, (%)** | |  |  |  |  |  |  |  |  |
| Paid work | 80.9  (76.0; 85.0) | 16.8  (11.5; 23.9) | 60.0  (54.4; 65.4) | 57.8  (47.2; 67.6) |  | 82.0  (76.2; 86.6) | 19.5  (13.1; 27.9) | 58.6  (49.1; 67.4) | 40.0  (22.3; 60.8) |
| Housework/Student | 19.1  (15.0; 24.0) | 83.2  (76.1; 88.5) | 40.0  (34.6; 45.6) | 42.2  (32.4; 52.8) |  | 18.0  (13.4; 23.8) | 80.5  (72.1; 86.9) | 41.4  (32.6; 50.9) | 60.0  (39.2; 77.7) |
| **Marital status, (%)** | |  |  |  |  |  |  |  |  |
| Married or cohabiting | 93.5  (90.0; 95.8) | 100  (100; 100) | 8.4  (6.0; 11.6) | 98.9  (92.3; 99.9) |  | 93.8  (89.7; 96.4) | 100  (100; 100) | 10.9  (6.6; 17.3) | 100  (100; 100) |
| **Delivery mode, (%)** | |  |  |  |  |  |  |  |  |
| Vaginal | 17.8  (13.8; 22.6) | 63.6  (55.4; 71.2) | 100  (100; 100) | 64.4  (53.9; 73.4) |  | 18.5  (13.8; 24.3) | 61.1  (51.7; 69.7) | 100  (100; 100) | 60.0  (39.2; 77.7) |
| ***Infant characteristics*** | | |  |  |  |  |  |  |  |
| **Sex, (%)** |  |  |  |  |  |  |  |  |  |
| Female | 51.2  (45.5; 56.9) | 49.7  (41.5; 57.9) | 45.2  (40.4; 50.2) | 45.1  (35.1; 55.5) |  | 50.2  (43.5; 57.0) | 51.3  (42.1; 60.5) | 49.7  (41.4; 57.9) | 48.0  (28.8; 67.8) |
| Male | 48.8  (43.1; 54.5) | 50.3  (42.1; 58.5) | 54.8  (49.8; 59.6) | 54.9  (44.5; 65.0) |  | 49.8  (43.0; 56.5) | 48.7  (39.5; 57.9) | 50.3  (42.1; 58.6) | 52.0  (32.2; 71.2) |
| **Gestational age in weeks, mean** | 38.9  (38.8; 39.1) | 39.1  (39.0; 39.3) | 39.2  (39.1; 39.4) | 39.0  (38.8; 39.3) |  | 38.9  (38.8; 39.1) | 39.1  (38.9; 39.3) | 39.4  (39.2; 39.6) | 39.0  (38.4; 39.5) |
| **Birthweight in grams, mean** | 3,212  (3,164; 3,259) | 3,094  (3,033; 3,155) | 3,188  (3,145; 3,231) | 2,886  (2,785; 2,987) |  | 3,213  (3,154; 3,272) | 3,087  (3,027; 3,147) | 3,164  (3,091; 3,237) | 2,905  (2,732; 3,078) |
| ***Feeding practices*** | |  |  |  |  |  |  |  |  |
| Exclusive breastfeeding at 3 months, (%) | - | - | - | - |  | 52.9  (45.0; 60.7) | 53.5  (43.6; 63.1) | 30.8  (21.4; 42.0) | 90.9  (68.1; 97.9) |
| Continued breastfeeding at 12 months, (%) | - | - | - | - |  | 52.3  (44.3; 60.1) | 87.2  (78.7; 92.7) | 75.7  (66.7; 82.8) | 100  (100; 100) |
| Minimum dietary diversity at 12 months, (%) | - | - | - | - |  | 88.9  (82.8; 93.0 | 46.8  (36.9; 57.0) | 61.1  (51.5; 69.9) | 88.2  (60.3; 97.4) |
